# Clinical Heterogeneity of Immune Dysregulation, Polyendocrinopathy, Enteropathy, X-Linked Syndrome: A French Multicenter Retrospective Study

**DOI:** 10.1038/s41424-018-0064-x

**Published:** 2018-11-02

**Authors:** R. Duclaux-Loras, F. Charbit-Henrion, B. Neven, J. Nowak, S. Collardeau-Frachon, C. Malcus, P. F. Ray, D. Moshous, J. Beltrand, O. Goulet, N. Cerf-Bensussan, A. Lachaux, F. Rieux-Laucat, F. M. Ruemmele

**Affiliations:** 1 Department of Paediatric GastroenterologyHepatology and Nutrition, Hospices Civils de Lyon, Hôpital Femme Mère Enfant, Bron, France; 2 Université Paris Descartes-Sorbonne Paris Cité, Paris, France; 3 INSERM, UMR1163, Laboratory of Intestinal Immunityand Imagine Institute, Paris, France; 4 Department of Pediatric, Gastroenterology Assistance Publique-Hôpitaux de Paris, Hôpital Necker-Enfants Malades, Paris, France; 5 Assistance Publique – Hôpitaux de Paris, Hôpital Necker-Enfants Malades, Paediatric Haemato-Immunology Unit, Paris, France; 6 Department of Pediatric Gastroenterology and Metabolic Diseases, Poznan University of Medical Sciences, Poznan, Poland; 7 Department of Pathology, Hospices Civils de Lyon, Hôpital Femme Mère Enfant, Bron, France; 8 Hospices Civils de Lyon, Hôpital Edouard Herriot, Laboratory of Immunology, Lyon, France; 9 Genetic Epigenetic and Therapies of Infertility, Institute for Advanced Biosciences, Inserm U1209, CNRS UMR 5309, Université Grenoble Alpes, 38000 Grenoble, France; 10 CHU de Grenoble, UF de Biochimie Génétique et Moléculaire, Grenoble, F-38000 France; 11 Assistance Publique – Hôpitaux de Paris, Hôpital Necker-Enfants Malades, Pediatric EndocrinologyDiabetology and Gynecology Department, Paris, France; 12 INSERM UMR 1163, Laboratory of Immunogenetics of Pediatric Autoimmune Diseases, Paris, France

## Abstract

**Objective:**

Immune dysregulation, polyendocrinopathy, enteropathy, X-linked (IPEX) syndrome is an autoimmune disease caused by mutations in the forkhead box protein 3 gene (*FOXP3*), which encodes a key regulator of immune tolerance. The aim of this study was to describe the clinical heterogeneity of the disease in a national French cohort.

**Methods:**

Multicenter retrospective study of patients diagnosed with IPEX syndrome caused by mutations in *FOXP3*.

**Results:**

Thirty children from 26 families were included. Age at disease onset (median [first to third quartile]) was 1.5 mo [0–84] and at death 3.5 years [0–10.5] (*n* = 15) indicating a high heterogeneity. Initial presentation was diarrhoea (68%), type 1 diabetes (T1D; 25%), skin lesions (7%) and nephropathy (3%). During the course of the disease the following main symptoms were observed: diarrhoea (100%), skin lesions (85%), T1DM (50%), severe food allergies (39%), haematological disorders (28%), nephropathies (25%), hepatitis (14%) as well as the presence of a variety of autoantibodies. Immunosuppressive mono- or combination therapy led to improvement in eight children. Three boys displayed a stable disease course without any immunosuppressive medication. Overall 10-year survival rate was 43% (42% in transplanted patients and 52% in patients on immunosuppressive therapy). Five out of 22 identified *FOXP3* mutations have not been described yet: c.−23 + 1G > A, c.−23 + 5G > A, c.264delC, c.1015C > T and c.1091A > G. The first two produced atypical, attenuated phenotypes. Missense and frameshift mutations affecting the forkhead domain were associated with poor survival (Gehan–Wilcoxon *p* = 0.002).

**Conclusion:**

The broad phenotypic heterogeneity of IPEX raises questions about modifying factors and justifies early *FOXP3* sequencing in suspected cases.

## Introduction

Immunodysregulation, polyendocrinopathy, enteropathy, X-linked syndrome (IPEX) is a rare monogenic primary immunodeficiency caused by mutations in forkhead box protein 3 (*FOXP3*) gene. In 1982, Powell et al.^1^ reported a large kindred with 19 affected males across 5 generations presenting diarrhoea, which was lethal in most infants in early childhood. In 2001, Chatila et al.^2^ identified mutations in *FOXP3* [initially called *JM2* in the centromeric region of the X chromosome (Xq11.3-q13.3)] in two unrelated families with IPEX phenotype. In mice, the so-called “scurfy mutation” which arose spontaneously in *Foxp3* and triggered a lymphoproliferative disease with multiorgan inflammation, has corroborated the causative role of FOXP3 in driving the disease^3^. The *FOXP3* gene is highly conserved across mammals and encodes a key transcription factor required for regulatory T cells (Tregs) development, maintenance and function^4^.

To date, approximately 150 patients carrying mutations in *FOXP3* gene have been reported. Classically, IPEX patients present multiorgan autoimmunity, including severe enteropathy, type 1 diabetes (T1D) and dermatitis. Outcome of patients is generally poor, unless successful hematopoietic stem cell transplantation (HSCT) can be proposed.

In the present retrospective multicentre French study of patients carrying *FOXP3* mutations, we aim to highlight the broad spectrum of symptoms in order to facilitate diagnosis as well as clinical management of this rare disease.

## Patients and Methods

### Patients

This multicentre retrospective study reviewed all IPEX patients treated at four French university hospitals between 1980 and 2015 (Necker-Enfants Malades Hospital—Paris, Lyon, Clermont-Ferrand and Bordeaux). Only patients with a documented mutation in the *FOXP3* gene were included. Recorded data comprised: age at onset, clinical symptoms (enteropathy, skin disease, endocrinopathy and allergy) and biological parameters (total IgE, autoimmune enteropathy-related—AIE75 kDa autoantibodies), endoscopic and histopathologic presentation, main therapeutics and long-term outcomes.

### Methods

Genomic DNA was isolated from peripheral blood using the QIAamp DNA Blood Mini Kit (Qiagen, Courtaboeuf, France). Eleven exons, including all intron–exon boundaries, were amplified from genomic DNA by means of PCR with specific intron-flanking primer pairs. Patients 1–20 and patient 30 were diagnosed at Necker Enfants-Malades Hospital in Paris and patient 21–29 were diagnosed in the university hospital in Grenoble as already described^5^.

For flow cytometry determination of Tregs, PBMCs were membrane stained with anti-CD4 and anti-CD25 monoclonal antibodies and then fixed, permeabilized, and stained with Alexa Fluor 488 anti-human FOXP3 monoclonal antibodies (used for patients 2, 4, 8, 14, 25 and 26) or allophycocyanin-labelled anti-human FOXP3 as described by Moes et al.^6^.

Consequences of mutations on protein function were predicted using three algorithms: Polyphen2, Sift (Sorting Intolerant From Tolerant, J. Craig Venter Institute) and Mutation Taster (www.mutationtaster.org). Mutations were next ranked on the basis of the predicted impact of each variant by combined annotation-dependent depletion (CADD), and compared with the mutation significance cutoff (MSC), a gene-level specific cutoff for CADD scores (http://pec630.rockefeller.edu:8080/MSC/).

We compared the survival of patients with forkhead domain-affecting and other *FOXP3* mutations for whom sufficient data were available using the Gehan’s generalised Wilcoxon test. This included patients who underwent HSCT or died in utero. We also employed Fisher’s test to investigate the proportions of patients surviving beyond the age of three years (the median of follow-up) depending on the presence or absence of this type of mutation (two-tailed *p* value reported). The age at onset in the two groups was compared using the Mann–Whitney *U* test. The alpha level was set at 0.05. Statistical analyses were performed using Statistica 12 (StatSoft Inc., Tulsa, USA).

## Results

### Demographic Data

Twenty-seven male infants, two brothers who died in utero (in the 19th and 24th week of gestation) and one preterm neonate (32nd week of gestation) from 26 families were included in this study (Table 1). None of the families were consanguineous. Nineteen patients have been previously described in cohort studies or as case reports^5–14^. The median age at disease onset was 1.5 month [first to third quartile; 0–84]. The median duration of follow-up was 4 years [0–22] and the average of age at last follow-up was 7.6 years.

**Table 1:** Characteristics of the French IPEX cohort

Pts	Mutation	CADD	Age at onset	Diar	Diab	Ecz	Other	AIE-75 kDa	Auto-immunity	Therapy	Complications	Follow-up	Reported by
1	g.−6247_−4859delGAG		3 wk	+	−	+	Allergy (food), cheilitis and sepsis	+	AEA	PN, Ctc, Aza, Rapa, FK506 Infliximab and HSCT	TM with ESRF pulmonary infection, acute pancreatitis, and osteopenia	Death 10.5 y	6, 10
2	g.−6247_−4859delGAG		5 wk	+	−	+	Allergy (food), cheilitis, sepsis and HP gastritis	+	AEA	Ctc, Aza, Rapa, Cyclo and FK506	Pulmonary adenoviral infection, BPNP and BD	Alive 14.5 y	6, 10
3	c.751_753 delGAG		4 wk	+	+	+	AHA, agranulocytosis and hepatitis	+	AEA, ANA, anti-islet, SMA, Coombs, anti-PNN and anti-GAD	PN, Ctc, Aza, FK506 and Rituximab	AHA, Pseudomonas infection and BD	Death 8 mo	6, 11
4	c.751_753 delGAG		6 wk	+	−	+	Hypothyroidism, interstitial nephritis and AHA	+	AEA	PN, Ctc, Aza, MTX, FK506, Rapa, Rituximab and HSCT	Rectal abscess, colostomy, cholangitis, EBV reactivation, BD, VZV infection, intracranial haemorrhage, cataract and bones fracture	Alive 17 y	6, 11
5	c.736 − 1G > C	24	7 wk	+	+	+	Allergy (food)	−	ANA, anti-insulin and anti-GAD	PN and Rapa	Pulmonary infection and BD	Alive 5 y	
6	c.816 + 5G > A	11	4 wk	+	+	+		+	AEA, anti-insulin and anti-enterocyte	PN, Ctc, Aza, Rapa and FK506	PNP and VZV infection wt hepatitis	Alive 14 y	6
7	c.1121T > G	27	4 wk	+	−	+	AHA, thrombocytopenia and allergy	+	na	Ctc, Aza, FK506 and Rapa	Death during HSCT induction therapy	Death 14 mo	6, 10, 11
8	c.1113T > G	23	4 wk	+	+	+	Anaemia	−	ANA and anti-GAD	PN, Ctc, FK506 and HSCT		Death 3 y	8
9	c.210delG		6 wk	+	−	+		+	na	PN, Ctc, FK506 and HSCT		Death 1.5 y	
10	c.1015C > G	24	1 wk	+	+	+	AHA	+	AEA	FK506	Septicaemia	Death 7 mo	11
11	c.1100T > G	27	3 mo	+	+	+	Tubulointerstitial nephritis	+	AEA, ANA, anti-GAD and anti-mitoch	Cts, Cyclo and ALS		Death 2 y	10
12	c.751_753 delGAG	27	1 wk	+	+	+	Membranous glomerulonephritis	na	AEA, ANA, SMA and anti-keratin	Cts, Cyclo and ALS	Recurrent infections (*S. hominis*. *S, epidermidis*)	Na	6
13	c.736 − 1G > A	24	8 wk	+	+	+	Anaemia, thrombocytopenia and membranous glomerulonephritis	na	AEA, ANA, anti-GAD, anti-mitoch and anti-platelet	Ctc, Cyclo and ALS	Recurrent infections (*S. epidermidis*, *S. aureus*)	Na	6
14	c.152G > A	20	10 mo	+	−	−		+	cANCA, ANA and anti-colonocyte	5-ASA, Ctc, Aza and FK506	/	Alive 4.5 y	
15	c.−23 + 1G > A	24	8 wk	+	−	+	Allergy (food++), anaemia and thrombocytosis	−		PN	Food allergy	Alive 3.3 y	
16	c.817A > C	5	7 y	+	−	+		+	AEA	Rapa	Interstitial nephritis secondary to Cyclo	Alive 10 y	9
17	c.1091A > G	26	24 days	+	−	+	Allergy (food)	+		PN, Aza and FK506		Alive 19 y	
18	c.264delC		5 y	+	+	−	Tubulo-interstitial nephritis, allergy (food)	+	Anti-GAD	Renal transplantation, Ctc, Rapa, MFM, FK506, ALS and HST	Diabetes under Ctc. exocrine pancreatitis, CMV and EBV reactivation, TM, Pseudomonas infection and digestive GvHD	Death 7.3 y	
19	c.1157G > A	34	4 wk	+	−	+	HMG with severe hepatitis, thrombocytopenia	na	na	Ctc, Rapa IV and Alemtuzumab	CMV infection and MRSA infection	Death 7 mo	
20	c.1010G > A	33	4 wk	+	+	−	Respiratory failure	+	Anti-GAD	PN, Rapa, Ins, noninvasive ventilation and HSCT	TM	Alive 1 y	
21	c.227delT		3 wk	+	−	+	Autoimmune hepatitis and membranous glomerulonephritis	+	Anti-actin, anti-TPO, ANA and AEA	PN, Ctc, Cyclo and Aza	Multiple flare-ups. and steroid-induced osteopenia	Alive 22 y	7, 12
22	c.227delT		2 wk	+	−	+		na	na	PN		Death 6 wk	7
23	c.816 +4A > G	7	8 wk	+	−	+	Cow’s milk protein allergy, asthma, SA and tubulointerstitial nephritis	+	Anti-actin	PN, Ctc, Cyclo and Aza	Septicaemia (*E. coli*)	Death 3 y	5
24	c.−23 + 5G > A	12	3 wk	+	+	+	Arthritis	+	Anti-thyroglobulin, ASCA and ANCA	PN and Ins		Alive 5 y	
25	c.-23 + 5G > A	12	13 mo	+	+	+		+	ASCA, anti-islet cell, anti-GAD and anti-TG	PN, Ins and Gluten eviction	Coeliac disease	Alive 3 y	
26	c.1189C > T	33	Birth	+	−	+		na	na	PN	Septicaemia (*E. coli*)	Death day 4	
27	c.1033C > T	24	IU	−	−	−		na	na	−		IFD	13
28	c.1033C > T	24	IU	−	−	−		na	na	−		IFD	13
29	c.751_753 delGAG		2 mo	+	−	+	Hypothyroidism	+	Anti-pancreas (exocrine) and anti-thyroglobulin	Rapa, Ctc and PN	*S. haemolyticus* sepsis	Alive 3 y	
30	c.1015C > T	25	4 wk	+	−	+	Cow’s milk protein allergy	+	AEA, ANA, anti-GAD, ANCA and anti-platelet	PN, Rapa IV, Ctc, Ruxo, Rituximab and HSCT	Pneumopathy complicated by septicaemia (*P. aeruginosa*)	Alive 6 mo	

*AEA* anti-enterocyte antibodies; *AHA* autoimmune haemolytic anaemia; *ALS* antilymphocyte serum; *ANA* antinuclear antibodies; *ANCA* antineutrophil cytoplasmic antibodies; *ASCA* anti-*Saccharomyces cerevisiae* antibodies; *anti-GAD* anti-glutamic acid decarboxylase antibodies; *anti-mitoch.* anti-mitochondria antibodies; *anti-TG* anti-transglutaminase antibodies; *anti-TPO* anti-thyroperoxidase antibody; *AZA* azathioprine; *BD* bronchial dilatation; *CADD* combined annotation-dependent depletion; *Ctc* corticoids; *Cyclo* cyclosporine; *Diar* diarrhoea; *Diab* diabetes; *Ecz* eczema; *ESRF* end-stage renal failure; *GvHD* graft versus host disease; *HMG* hepatomegaly; *HP* *Helicobacter pylori*; *HSCT* hematopoietic stem cell transplantation; *IFD* intrauterine foetal death; *Ins* insulin; *IV* intravenous; *MFM* mycophenolate mofetil; *mo* month; *MRSA* methicillin-resistant *Staphylococcus aureus*; *MTX* methotrexate; *na* not available; *PN* parenteral nutrition; *Rapa* rapamycin; *Ruxo* ruxolitinib; *SMA* smooth muscle antibodies; *TM* thrombotic microangiopathies; *wk* week; *y* year.

### Clinical and Biological Data

Chronic diarrhoea was the most frequent symptom: it was the initial symptom in 18 patients (68%) and was present in 28 patients (100%) during the course of the disease (Table 1). Skin lesions were mainly eczematous and were associated with diarrhoea in 22 patients (78%). Erythroderma was the first and main symptom in patients (pts) 7 and 26. In seven patients, the first clinical manifestation was T1D, at a median age of 1.5 months [0–13 months]. Twelve patients (43%) developed T1D during the course of the disease. Nine of them (75%) had positive anti-glutamic acid decarboxylase or anti-islet antibodies.

Ten patients (35%) developed severe food allergy. The main incriminated food antigen was cow’s milk protein, with presence of specific IgE in all tested patients. Among these patients, pt15 presented at the age of 2 months with severe diarrhoea associated with cow’s milk protein allergy. Although diarrhoea resolved spontaneously, his food allergy extended to eggs, exotic fruits, nuts, soy, beef and veal without any other intestinal or extra-intestinal symptoms.

Haematological disorders occurred in eight patients (29%), six had anaemia (positive Coomb’s test *n* = 4), three had thrombocytopenia (anti-platelet antibodies *n* = 2) and one had neutropenia with antineutrophil antibodies.

Eight patients (25%) developed nephropathy (median age 3 years [1.8–9]). Interstitial nephropathy was described in five cases (pts 4, 11, 16, 18 and 23) among them, in three most probably linked to cyclosporine medication (pts 11, 16 and 23). Three patients presented glomerulonephritis linked to the disease (pts 12, 13 and 21). Chronic nephritis was the initial manifestation in pt 18; it led to chronic renal failure that required peritoneal dialysis at the age of five followed by kidney transplantation at the age of six.

Four (14%) patients developed hepatitis, with presence of anti-smooth muscle autoantibodies in two cases (pts 3 and 21). One case of hepatitis occurred during mercaptopurine therapy (pt 17).

**Fig. 1:**
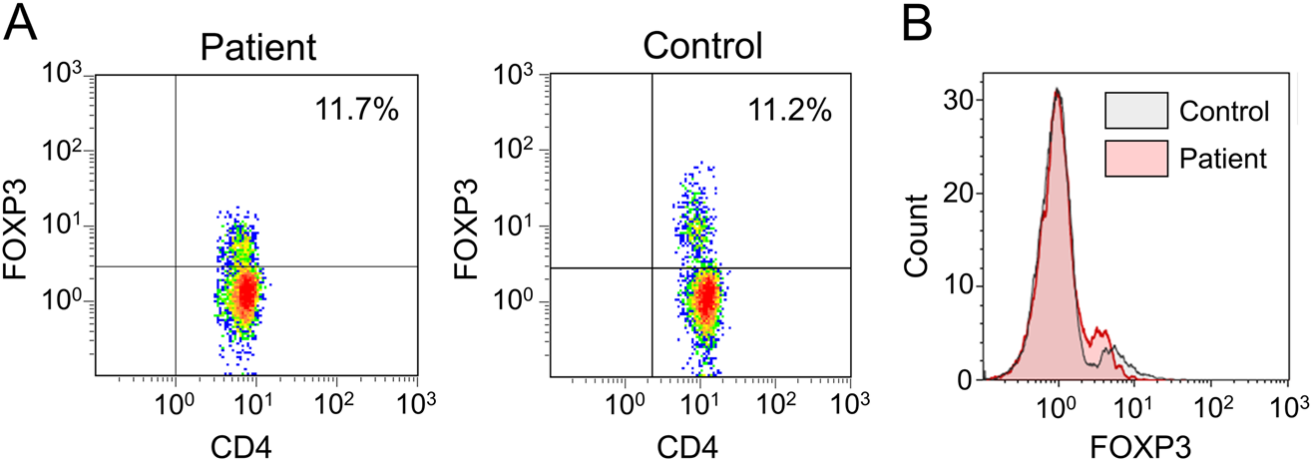
Evaluation of regulatory T cells frequency in an atypical IPEX case. **a** Flow cytometry on isolated PBMCs from patient 25 and a healthy control subject after membrane staining with CD3 and CD4 and intracellular staining of FOXP3. **b** Mean fluorescence intensity of FOXP3 in CD3^+^CD4^+^ cells in control and patient 25

Twenty-one out of 23 tested patients (91%) had markedly elevated IgE, ranging between 94 and 12,000 kUI/L (*N* < 40 kUI/l). Autoantibodies to AIE 75 kDa, directed against harmonin, a brush border protein^15^, were found in 22 out of the 23 tested patients (95%). Twenty patients showed positive auto-antibodies against various targets (summarised in Table 1). Among them, 13 patients showed positive anti-enterocyte antibodies. No auto-antibody was detected in two patients (pts 15 and 17). Peripheral Tregs were analysed in ten subjects. In three children (pts 1, 2 and 3), CD4^+^CD25^+^FOXP3^+^ Tregs represented less than 1% of peripheral CD4^+^ T (normal range: 5–10%). Patients 8, 10 and 21 had reduced levels 3.0%, 3.3% and 1.5%, respectively. In contrast, Tregs numbers were normal in pts 7 (8.0%), 24 (7.5%), 25 (11.0%) and 30 (7.5%), while the mean of fluorescence was decreased in pt 25 (Fig. 1).

**Fig. 2:**
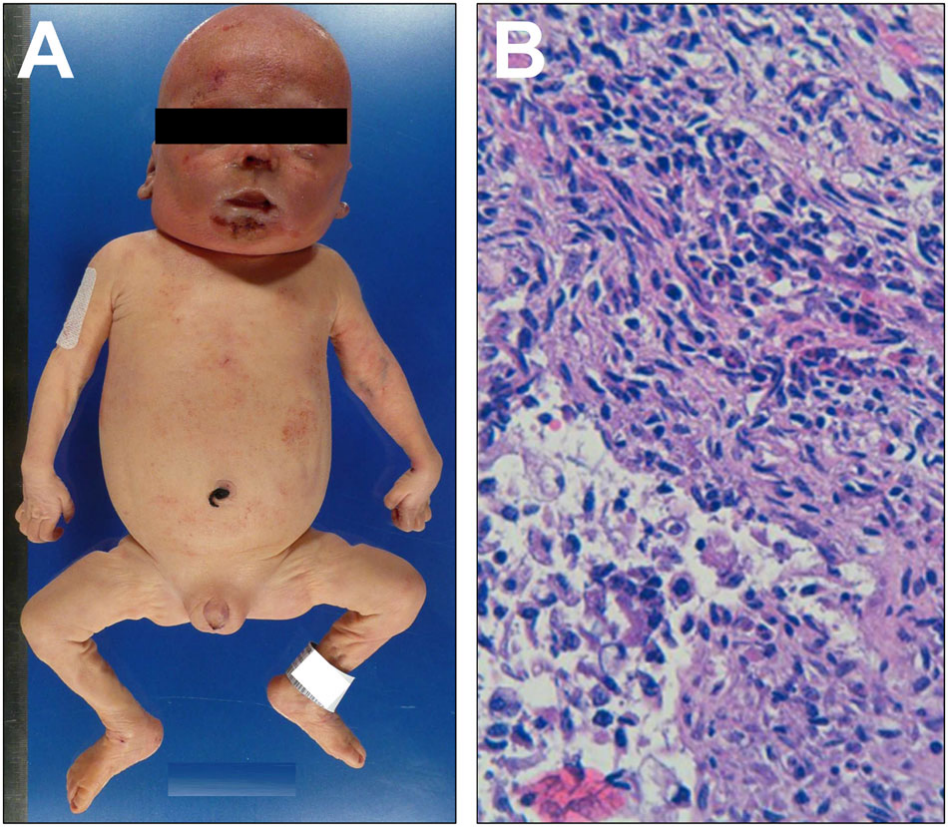
Histological analysis of neonatal IPEX form. **a** Standard autopsy picture of patient 26, showing diffuse skin erythema and **b** haematoxylin–eosin standard staining of an intestinal biopsy showing mononuclear infiltrate of the intestinal chorion

Three patients with atypical phenotypes were included (pts 15, 24 and 25). Patients 15 and 24 presented severe diarrhoea early in their life, which resolved without immunosuppressive therapy. One is now 4-year-old with a severe food allergy and the second has T1D. Pt 25, a cousin of pt 24 presented with late onset diabetes at the age of 13 months without gastro-intestinal tract involvement. He later developed a “coeliac-like” disease with positive anti-transglutaminase antibody and severe villous atrophy. Despite high AIE-75 kDa, hypereosinophilia and elevated total IgE pts 24 and 25 had a normal frequency of Tregs (Fig. 2).

### Endoscopic and histological data

Nineteen patients underwent at least one endoscopic procedure during the course of the disease. Six patients presented only inflammation in the upper gastro-intestinal tract with pale and fragile mucosa (pts 3, 6, 7, 8, 9 and 23). Patient 25 presented severe duodenal villous atrophy associated with high level of anti-transglutaminase. Seven patients had a combination of upper gastrointestinal and colon inflammation with severe inflammation and ulcerations at onset of symptoms (pts 1, 2, 4, 16, 19, 21 and 24). Patient 14 displayed only colitis and pt29 an ileocolitis. Endoscopy showed normal mucosa in pt5 despite severe diarrhoea, for pt18, who was under immunosuppressive therapy in the context of a pre-transplant work-up. Also, no macroscopic inflammation was reported for pt15 during a classical evaluation of the disease course.

Duodenal biopsies showed polymorphic infiltration of the lamina propria with lymphocytes, macrophages, plasmocytes and eosinophils. Histologic analysis of the lower part of the gut (colon sigmoid and rectum) highlighted an inflammatory infiltrate of the lamina propria with a predominance of lymphocytes and eosinophils.

Autopsy of pt26, who died in the neonatal period, showed CD3^+^CD4^+^ lymphocytic inflammatory infiltrates of the skin, the digestive tract, the pancreas and of the liver as well (Fig. 2).

### *FOXP3* mutations

Molecular diagnosis was obtained by targeted Sanger sequencing. Median age at molecular diagnosis was 5 years [0.25–8] and 4 years for patients born after 2001 (the discovery of *FOXP3* as disease causing mutation).

**Fig. 3:**
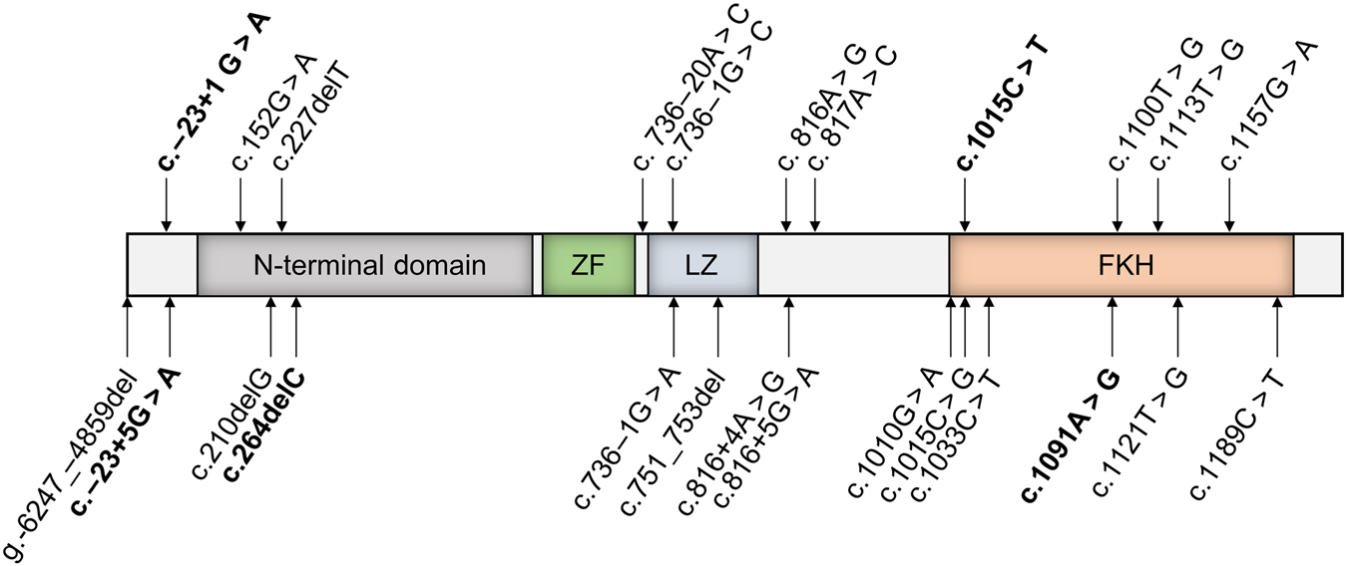
*FOXP3* mutations in the cohort. Schematic representation of the *FOXP3* gene with all the mutations found in our IPEX patients indicated

As shown in Fig. 3, 22 germinal mutations were identified in our cohort. Amongst these variants, four novel missense mutations were identified: c.−23 + 1G > A (pt 15), c.−23 + 5G > A (pts 24 and 25) c.1015C > T (pt30), c.1091A > G (pt17) and one nucleotide deletion mutation c.264delC (pt18), which resulted in a frameshift and introduced a premature stop codon. The first two variants were designated as splice-altering by in silico splicing defect predictions tools while they were all absent from the Exome Aggregation Consortium (ExAC) database. Of note, the CADD of c.−23 + 1G > A (24), c.−23 + 5G > A (12), c.1015C > T (25) and c.1091A > G (26) were well above the MSC of FOXP3 (0.002) (Table 1).

### Treatment and outcome

**Fig. 4:**
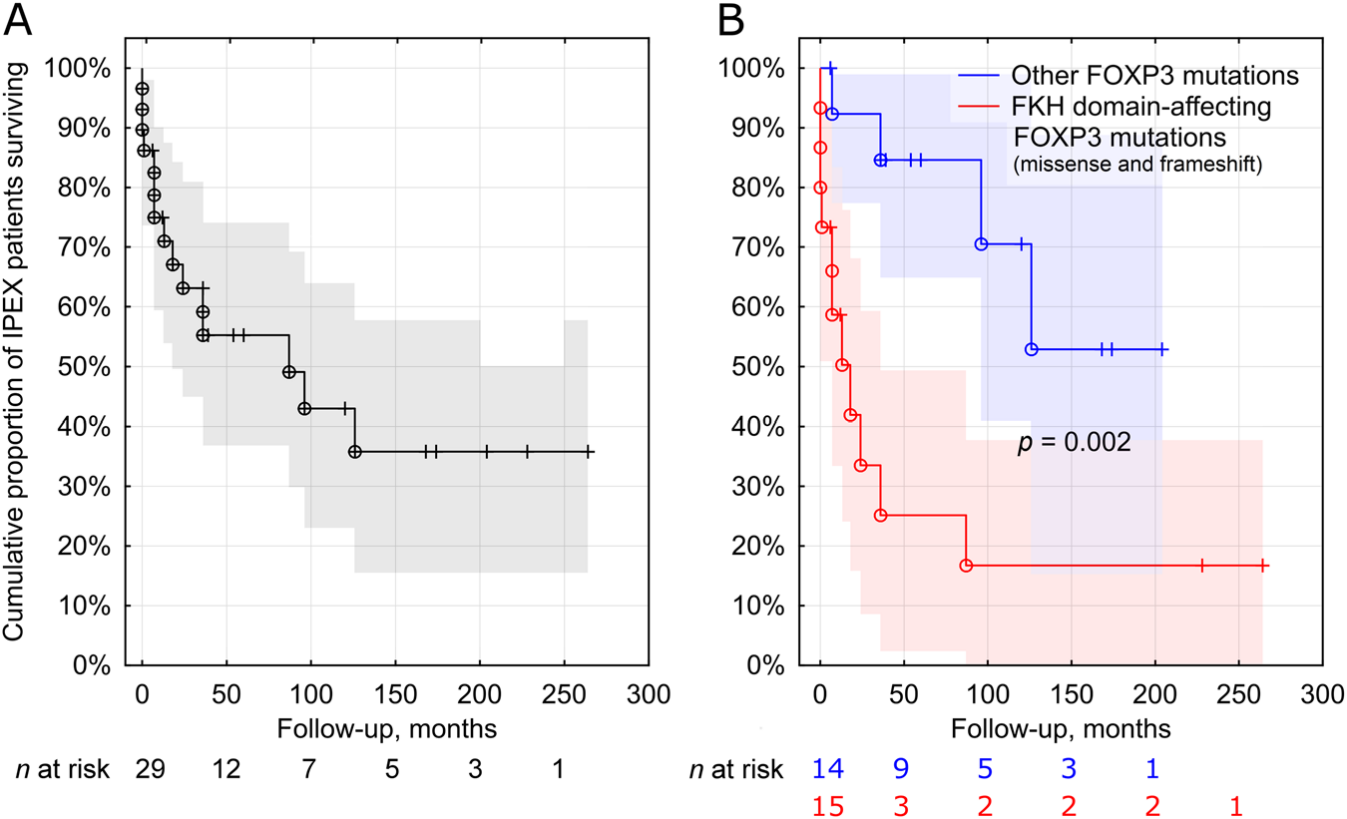
**a** Survival of IPEX patients in the French cohort. **b** Kaplan–Meier curves illustrating the poorer survival of IPEX patients harbouring forkhead (FKH) domain-affecting *FOXP3* mutations as compared with other mutations

The mean follow-up was 5 years (range: 0–22), two patients were lost to follow-up, and 10-year survival was 43% (Fig. 4a). Twenty-three patients received steroids. During the course of the disease, 13 patients took azathioprine and 2 were treated with mycophenolate mofetil. Five patients died during the first year of life due to multiorgan failure caused by severe diarrhoea (pts 3, 7, 10, 19, 22 and 25), at a median age of 5 months (0–9). Three patients died during a flare-up of their disease or due to *Escherichia coli* septicaemia and multiple organ failure (pts 9, 11 and 23). Three patients improved on tacrolimus (pts 6, 14 and 16) and four on sirolimus medication (pts 2, 5, 20 and 30). Among them two were also receiving azathioprine (pts 2 and 6). One patient was treated by cyclosporine, mycophenolate mofetil and steroids (pt 21). Three patients never received immunosuppressive therapy (pts 15, 24 and 25).

Seven patients underwent HSCT at a mean age of 5.5 years (range: 0.5–12.8) (Table 2). Two patients were transplanted with a matched sibling donor, three patients (pts 9, 18 and 30) with a mismatched related donor and two patients (pts 1 and 20) with a matched unrelated donor. Conditioning regimens (CR) are given in Table 2. Five patients displayed full engraftment, one patient transplanted with a non-myeloablative CR showed mixed chimerism, while one patient (pt9) failed to engraft. Four patients died after HSCT, due to graft-versus-host disease and transplant-related toxicities (microangiopathies and renal failure) (*n* = 2), graft rejection (*n* = 1) or hemophagocytosis at 30 months post-transplant (pt8).

**Table 2:** Outcomes in IPEX patients treated by hematopoietic stem cell transplantation

Pt	Age at onset	Age at BMT	Mutation	Conditionning	Donor	GvH prevention	Remission after transplantation	GvHD	Complication post after transplantation	Outcome
1	1 mo	10.5	g.-6247_-4859 delGAG	Flu, ALM and Bu (RIC)	MUD	Steroids/MMF	/	Acute grade II	Microangiopathies and renal failure	Death 10.5 y
4	1.5 mo	12.8	c.751_753 delGAG	Flu, ALM and Bu (RIC)	MSD	CY/MMF	Yes	Absence		Alive 17 y
8	1 mo	0.5	c.1113T > G	ATG, CY and Bu (MAC)	MSD	CY IV	Yes	Absence	Hemophagocytosis	Death 3 y
9	1.5 mo	1	c.210delG	ATG, BU and CY (MAC)	MMRD	CY/MMF	/	Absence	Graft rejection	Death 1.5 y
18	5 y	7	c.264delC	flu, Cx, TBI 2GY and post CY (NMA)	MMRD	CY/MMF	/	Grade III	Microangiopathies and renal failure	Death 7.3 y
20	1 mo	1	c.1010G > A	flu, Bu and ALM	MUD	CY/MMF	Yes	Grade III		Alive 2 y
30	1 mo	5.5	c.1015C > T	Ritu, ALM, Bu, Flu and post CY (MAC)	MMRD	CY/MMF	Yes	Chronic		Alive 1.5 y

*ALM* alemtuzumab; *ATG* anti-thymoglobulin; *Bu* busulfan; *CY* cyclophosphamide; *post CY* post-transplant cyclophosphamide; *BM* bone marrow; *Flu* fludarabine; *RIC* reduced intensity conditioning; *MAC* myeloablative conditioning; *NMA* non myeloablative conditioning; *GvHD* graft-versus-host disease; *Melph* melphalan; *mo* month; *MSD* matched sibling donor; *MUD* matched unrelated donor; *MMRD*: mis-matched related donor, *Pts* patients; *TBI* total body irradiation; *y* year, *MMF* mycophenolate mofetil; *TM* thrombotic microangiopathies; *ESRF* end-stage renal failure

The survival of children harbouring forkhead domain-affecting mutations (both missense and frameshift; *n* = 15) was poorer than that of children with other mutations (*n* = 14; *p* = 0.002; Fig. 4b). Amongst the 12 patients who died before the age of three, 10 harboured a forkhead domain-affecting mutation (83.3%) (*p* = 0.005). Only three patients with a similar mutation survived beyond the age of three years (23.1%) (*p* = 0.005). The age at onset of the disease did not differ between the two groups (*p* = 0.14).

## Discussion

Our national multicentre cohort of 30 IPEX patients unravels an enlarged clinical spectrum of IPEX with atypical phenotypes, and mild evolutions in some patients even without therapy. These observations are particularly important when defining the most appropriate treatment strategy while looking for genetic prognostic factor.

In 2012, Barzaghi et al. comprehensively reviewed all 136 cases described in the previous 12 years with the aim to define the clinical features of the syndrome^16^. The major elements of the clinical picture were: diarrhoea, T1D, and skin involvement. In our study the prevalence of all three main symptoms was in keeping (less than 15% difference) with this previous report^16^. Diarrhoea was the leading manifestation, present in 100% of patients and often life-threatening. Eczematous skin lesions concerned nearly three-quarters of cases. Almost half of the children had T1D; in most of them it was the first manifestation of IPEX. One in five subjects had renal disorders. Of particular interest, we observed a large percentage of food allergic patients in our cohort, 35% compared to 11% in the previous report. We suggest that because of a high rate of allergic symptoms, clinicians should carefully consider the introduction of complementary foods in IPEX patients.

Our study described several unusual phenotypes. Patient 18 presented first with renal tubulo-interstitial nephritis, which required renal transplantation and he developed classical severe diarrhoea only 1 year after organ transplant. A 7-month-old baby boy who exhibited a glomerulonephritis in the absence of any digestive problems was also previously reported, suggesting indeed that renal disorder could be the first manifestation of the disease^17^. Mutations in the FKH domain interfere with nuclear import and DNA binding, both of which are critical for FOXP3 repressor activity. These latter mutations may also be associated with more attenuated phenotypes.

Our cohort includes three patients with atypical phenotypes (pts 15, 24, and 25). Despite severe diarrhoea early in life, none of them needed immunosuppressive therapy. Two novel mutations were identified in the three patients: c.−23 + 1G > A and c.−23 + 5G > A. Interestingly, in pt 25 a decreased FOXP3 expression was observed on CD3^+^CD4^+^FOXP3^+^ cells compared to control despite normal frequency of Tregs (Fig. 1). All three patients (15, 24 and 25) carry intronic mutations located within the first splice donor site. Mutations outside *FOXP3* coding regions, within an intron/exon splice junction or in the first polyadenylation signal of the gene, may interfere with normal gene expression and protein production. Moreover, it has been shown the first splice donor site is highly methylated due to the presence of multiple conserved noncoding enhancer sequences, responsible of epigenetic regulation^18^. As they are both hypomorphic mutations, we speculate that variants in this particular region might affect the overall methylation status leading to a decreased FOXP3 expression and resulting in an atypical phenotype.

We also report a new IPEX case of a preterm infant with severe erythroderma (pt26) who died of septicaemia at day four of life. Although autoimmune diseases in utero are very rare, the number of reported neonatal cases of IPEX has been increasing since the first description in 2015 (13). Foetal and neonatal forms of IPEX indicate that the disease could start in utero, which suggests that Tregs are important for foetal tolerance.

A large variety of autoantibodies are described in IPEX. Anti-harmonin (anti-AIE75 kDa) is the most specific antibody in IPEX-patients, even though it has also been described in CD25 deficiency^15^. Totally, 90% of patients described herein had a positive anti-AIE75 kDa serology. Therefore, we find it to be a useful tool for the preliminary screening of patients that may possibly distinguish between IPEX syndrome and other immune deficiencies, such as autoimmune polyendocrinopathy–candidiasis–ectodermal dystrophy^19^. In our experience, severity and evolution of the disease do not correlate with the titre of anti-AIE75 kDa. In four of our patients who previously had positive anti-AIE75 kDa titres the antibodies have become undetectable under therapy. Indeed, it was shown that anti-AIE75 kDa in IPEX patients become undetectable or persist at low titres in remission after immunosuppressive therapy or HSCT^20^.

The largest yet still homogenous group of pathogenic variants that we could identify were mutations affecting the forkhead domain of *FOXP3*. In our cohort, the survival of patients with variants of this type was markedly poorer and rarely exceeded three years. This highlights its potential use as a genetic prognostic factor in IPEX. Our exploratory search for associations between phenotypic traits and forkhead domain-affecting *FOXP3* mutations yielded negative results. The lack of genotype–phenotype correlation may reflect the complex intracellular interactions of FOXP3, and strongly suggests the role of epigenetic regulation in determining the clinical picture and outcome (18). The range of manifestations and severity can also vary between patients with the same mutation, suggesting that modifier loci in the genetic background, as HLA haplotype and the lymphocyte repertoires, and/or environmental exposures modify the course of disease.

Lack of Tregs lead indirectly to a deregulation of T effector with an enrichment of T helper type 2 cells, an over production of IL-17 cytokines and a deregulation of autoreactive B cells (21). This balance between regulatory cells and effector cells could change from a patient to another.

The two main treatment modalities for IPEX syndrome are chronic immunosuppression and hematopoietic stem cell transplantation. Immunosuppressive therapy has to be introduced as soon as possible in order to limit any further damage. In our study, 25 patients received steroids, which appear to be the most efficacious first-line pharmacotherapy, then followed by other immunosuppressive agents for long-term therapy. As observed in this series, combination of at least two immunosuppressive drugs is needed to control the disease. In our cohort, only two patients improved on tacrolimus alone and three on sirolimus alone. We made a greater use of azathioprine with tacrolimus compared with previous reports, where mycophenolate mofetil was a common choice. Classically, a top-down approach with initial steroids and sirolimus is advised (approx. 0.15 mg/kg/day), adjusted to maintain serum sirolimus levels between 8 and 12 ng/mL^21^. In the majority of cases, chronic immunosuppression is partially effective, and increases the risks of severe and/or opportunistic infections.

HSCT is currently the only curative therapy for IPEX. HSCT was initially proposed after multiple relapses and when combination immunosuppressive therapy had been tried and failed. Early HSCT seems to lead to better outcomes. Fifteen IPEX patients treated by HSCT were described so far in the literature. Most of them (10/15) received the transplant in their first two years of life with a large survival rate (90%). A recent study by Nademi et al.^22^ also supports this observation: of five infants who had HSCT in the first year of life (two at six months and three at ten months) all patients survived. In our patients the median age at HSCT was 5.5 (range: 0.5–12.8) years.

In conclusion, IPEX is phenotypically heterogeneous ranging from severe intrauterine forms to moderate phenotypes with atypical course of the disease which do not require extensive immunosuppressive treatment. Early *FOXP3* sequencing facilitates rapid diagnosis, which is necessary since it permits aggressive, combined immunosuppressive therapy and opens the way to early HSCT.

## Study Highlights

### What is current knowledge?

- Immune dysregulation, polyendocrinopathy, enteropathy, X-linked (IPEX) syndrome is an autoimmune genetic disorder.
- Main clinical features include severe refractory enteropathy, type 1 diabetes and dermatitis.
- Hematopoietic stem cell transplantation is the only known curative treatment.

### What is new here?

- *FOXP3* mutations localised in the first intron can produce atypical, attenuated phenotypes.
- Mutations affecting the forkhead domain are associated with poor survival.
- Five new FOXP3 mutations leading to IPEX phenotype are identified: c.−23 + 1 G > A, c.−23 + 5G > A, c.264delC, c.1015C > T and c.1091A > G.
- Immunosuppressive therapy may yield excellent results in selected patients.
